# 3-Hy­droxy-*N*′-[(*E*)-2-thienyl­methyl­idene]-2-naphtho­hydrazide

**DOI:** 10.1107/S160053681005405X

**Published:** 2011-01-08

**Authors:** Qingkun Wu, Handong Yin, Daqi Wang

**Affiliations:** a College of Chemistry and Chemical Engineering, Liaocheng University, Shandong 252059, P.R.China

## Abstract

The asymmetric unit of the title compound, C_16_H_12_N_2_O_2_S, contains three independent mol­ecules. Intra­molecular N—H⋯O hydrogen bonds in the three mol­ecules lead to very similar conformations: the thio­pene ring and naphthalene ring system in the three mol­ecules form dihedral angles of 10.3 (2), 9.1 (2) and 9.3 (3)°. In the crystal structure, inter­molecular O—H⋯O hydrogen bonds link the mol­ecules into chains propagating in [031].

## Related literature

For related structures, see: Huang (2009 ▶); Liang *et al.* (2008 ▶); Shafiq *et al.* (2009 ▶).
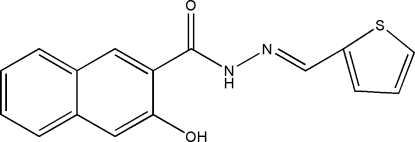


## Experimental

### 

#### Crystal data

C_16_H_12_N_2_O_2_S
*M* *_r_* = 296.34
Orthorhombic, 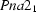

*a* = 19.8191 (16) Å
*b* = 6.2487 (5) Å
*c* = 33.947 (3) Å
*V* = 4204.1 (6) Å^3^
*Z* = 12
Mo *K*α radiation
μ = 0.24 mm^−1^
*T* = 298 K
0.41 × 0.17 × 0.06 mm

#### Data collection

Bruker SMART 1000 CCD area-detector diffractometer
Absorption correction: multi-scan (*SADABS*; Sheldrick, 1996 ▶) *T*_min_ = 0.909, *T*_max_ = 0.986
20887 measured reflections
7258 independent reflections
3449 reflections with *I* > 2σ(*I*)
*R*_int_ = 0.077

#### Refinement

*R*[*F* ^2^ > 2σ(*F* ^2^)] = 0.055
*wR*(*F* ^2^) = 0.078
*S* = 0.97
7258 reflections
569 parameters
11 restraints
H-atom parameters constrained
Δρ_max_ = 0.28 e Å^−3^
Δρ_min_ = −0.24 e Å^−3^
Absolute structure: Flack (1983 ▶), 3473 Friedel pairs
Flack parameter: 0.01 (7)

### 

Data collection: *SMART* (Bruker, 2007 ▶); cell refinement: *SAINT* (Bruker, 2007 ▶); data reduction: *SAINT*; program(s) used to solve structure: *SHELXS97* (Sheldrick, 2008 ▶); program(s) used to refine structure: *SHELXL97* (Sheldrick, 2008 ▶); molecular graphics: *SHELXTL* (Sheldrick, 2008 ▶); software used to prepare material for publication: *SHELXTL*.

## Supplementary Material

Crystal structure: contains datablocks I, global. DOI: 10.1107/S160053681005405X/cv5019sup1.cif

Structure factors: contains datablocks I. DOI: 10.1107/S160053681005405X/cv5019Isup2.hkl

Additional supplementary materials:  crystallographic information; 3D view; checkCIF report

## Figures and Tables

**Table 1:** Hydrogen-bond geometry (Å, °)

*D*—H⋯*A*	*D*—H	H⋯*A*	*D*⋯*A*	*D*—H⋯*A*
O6—H51⋯O2	0.82	2.03	2.771 (5)	149
N6—H54⋯O6	0.86	1.96	2.641 (5)	135
N2—H2*A*⋯O4	0.86	1.96	2.660 (5)	138
N4—H53⋯O5	0.86	1.90	2.614 (5)	139
O5—H50⋯O1	0.82	1.87	2.682 (5)	169
O4—H4⋯O3^i^	0.82	2.40	2.688 (5)	102

Symmetry code: (i) 
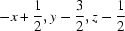
.

**Fig. 1.**
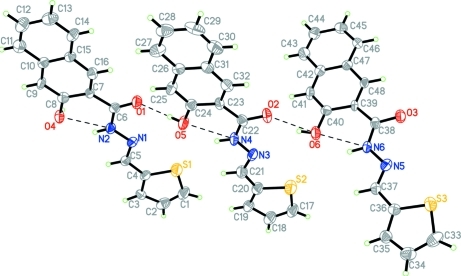
Three independent molecules of (I) showing the atomic numbering and 50% probability displacement ellipsoids. The hydrogen bonds are shown as dashed lines.

## supplementary crystallographic information

### Comment

In continuation of our research of organotin derivatives, the title compound (I) - a novel Schiff base - have been synthesized. Herewith we present its crystal structure.

In (I), there are three independent molecules in the asymmetric unit (Fig. 1). All the bond lengths and angles are normal and correspond to those observed in the related compounds (Huang (2009); Liang *et al.* (2008); Shafiq *et al.* (2009). Intramolecular N—H···O hydrogen bonds (Table 1) lead to very close conformations - the thiopene ring and naphthalene bicycle in three molecules form dihedral angles of 9.67 (27)°, 9.16 (29)° and 9.41 (26)°, respectively.

In the crystal structure, intermolecular O—H···O hydrogen bonds  (Table 1) link the molecules into chains propagated in direction [031].

### Experimental

The methanol solution of thiophene(0.1 mol) was added dropwise to a solution of 3-hydroxyl-2-naphthohydrazide(0.1 mol) in mehtanol. Then the mixture was stirred at room temperature for 6 h, during which time a yellow precipitate can be observed. The mixture was filtrated and the obtained solid was recrystallized from methanol. Yield 80%. *M*.p. 245°C. Anal. Calcd (%) for C_16_H_12_N_2_O_2_S (Mr = 296.34): C, 64.84; H, 4.08; N, 9.45. Found (%): C, 64.82; H, 4.03; N, 9.49.

### Refinement

All H atoms were placed in geometrically idealized positions (C—H=0.93 Å, O—H=0.82 Å, N—H=0.86 Å) and refined using a riding model, with *U*_iso_(H) = 1.2 *U*_eq_(C, O, N).

### Crystal data

**Table:**

C_16_H_12_N_2_O_2_S	*D*_x_ = 1.405 Mg m^−^^3^
*M**_r_* = 296.34	Mo *K*α radiation, λ = 0.71073 Å
Orthorhombic, *P**n**a*2_1_	Cell parameters from 2181 reflections
*a* = 19.8191 (16) Å	θ = 2.4–26.4°
*b* = 6.2487 (5) Å	µ = 0.24 mm^−^^1^
*c* = 33.947 (3) Å	*T* = 298 K
*V* = 4204.1 (6) Å^3^	Block, yellow
*Z* = 12	0.41 × 0.17 × 0.06 mm
*F*(000) = 1848	

### Data collection

**Table:**

Bruker SMART 1000 CCD area-detector diffractometer	7258 independent reflections
Radiation source: fine-focus sealed tube	3449 reflections with *I* > 2σ(*I*)
graphite	*R*_int_ = 0.077
phi and ω scans	θ_max_ = 25.0°, θ_min_ = 2.1°
Absorption correction: multi-scan (*SADABS*; Sheldrick, 1996)	*h* = −23→23
*T*_min_ = 0.909, *T*_max_ = 0.986	*k* = −7→6
20887 measured reflections	*l* = −40→38

### Refinement

**Table:**

Refinement on *F*^2^	Secondary atom site location: difference Fourier map
Least-squares matrix: full	Hydrogen site location: inferred from neighbouring sites
*R*[*F*^2^ > 2σ(*F*^2^)] = 0.055	H-atom parameters constrained
*wR*(*F*^2^) = 0.078	*w* = 1/[σ^2^(*F*_o_^2^) + (0.0055*P*)^2^] where *P* = (*F*_o_^2^ + 2*F*_c_^2^)/3
*S* = 0.97	(Δ/σ)_max_ < 0.001
7258 reflections	Δρ_max_ = 0.28 e Å^−^^3^
569 parameters	Δρ_min_ = −0.24 e Å^−^^3^
11 restraints	Absolute structure: Flack (1983), 3473 Friedel pairs
Primary atom site location: structure-invariant direct methods	Flack parameter: 0.01 (7)

### Fractional atomic coordinates and isotropic or equivalent isotropic displacement parameters (Å^2^)

**Table:**

	*x*	*y*	*z*	*U*_iso_*/*U*_eq_	
C17	0.1250 (3)	1.3649 (9)	0.3320 (2)	0.0772 (16)	
H17	0.1041	1.4728	0.3465	0.093*	
C18	0.1214 (3)	1.3474 (9)	0.2938 (2)	0.0852 (18)	
H18	0.0986	1.4477	0.2785	0.102*	
C19	0.1535 (2)	1.1707 (8)	0.27682 (17)	0.0632 (15)	
H19	0.1533	1.1341	0.2503	0.076*	
C20	0.1862 (3)	1.0582 (9)	0.30730 (18)	0.0638 (14)	
C21	0.2286 (3)	0.8714 (9)	0.30409 (19)	0.0645 (17)	
H21	0.2388	0.8180	0.2792	0.077*	
C22	0.3225 (3)	0.4907 (9)	0.35439 (19)	0.0496 (14)	
C23	0.3623 (3)	0.2970 (8)	0.34149 (16)	0.0469 (14)	
C24	0.3623 (3)	0.2105 (8)	0.30317 (18)	0.0533 (15)	
C25	0.3998 (3)	0.0317 (8)	0.29514 (19)	0.0615 (17)	
H25	0.3989	−0.0254	0.2698	0.074*	
C26	0.4393 (3)	−0.0666 (9)	0.32362 (19)	0.0524 (16)	
C27	0.4776 (3)	−0.2522 (10)	0.3153 (2)	0.076 (2)	
H27	0.4769	−0.3107	0.2901	0.091*	
C28	0.5149 (3)	−0.3436 (11)	0.3434 (2)	0.084 (2)	
H28	0.5397	−0.4657	0.3375	0.101*	
C29	0.5173 (3)	−0.2589 (13)	0.3819 (2)	0.092 (2)	
H29	0.5430	−0.3244	0.4014	0.111*	
C30	0.4813 (3)	−0.0799 (11)	0.3897 (2)	0.078 (2)	
H30	0.4836	−0.0205	0.4148	0.094*	
C31	0.4410 (3)	0.0179 (10)	0.36158 (19)	0.0547 (16)	
C32	0.4021 (3)	0.1999 (9)	0.36920 (17)	0.0573 (16)	
H32	0.4032	0.2579	0.3944	0.069*	
C33	0.4184 (3)	1.8592 (9)	0.5359 (2)	0.080 (2)	
H33	0.4346	1.9553	0.5545	0.096*	
C34	0.4305 (3)	1.8749 (10)	0.4964 (2)	0.0744 (19)	
H34	0.4572	1.9798	0.4848	0.089*	
C35	0.3980 (2)	1.7149 (9)	0.47596 (17)	0.0592 (16)	
H35	0.3994	1.7044	0.4486	0.071*	
C36	0.3635 (2)	1.5730 (8)	0.49900 (16)	0.0495 (14)	
C37	0.3271 (3)	1.3905 (8)	0.48448 (16)	0.0524 (15)	
H37	0.3261	1.3596	0.4577	0.063*	
C38	0.2277 (3)	0.9641 (9)	0.51485 (17)	0.0455 (15)	
C39	0.1955 (2)	0.7777 (8)	0.49573 (14)	0.0381 (13)	
C40	0.2039 (2)	0.7167 (8)	0.45615 (16)	0.0461 (14)	
C41	0.1721 (3)	0.5405 (9)	0.44212 (16)	0.0573 (16)	
H41	0.1794	0.5006	0.4161	0.069*	
C42	0.1280 (3)	0.4142 (8)	0.46550 (17)	0.0492 (15)	
C43	0.0939 (3)	0.2328 (9)	0.45136 (18)	0.0686 (18)	
H43	0.0990	0.1920	0.4252	0.082*	
C44	0.0541 (3)	0.1183 (9)	0.47519 (19)	0.0651 (17)	
H44	0.0310	0.0003	0.4654	0.078*	
C45	0.0470 (2)	0.1750 (9)	0.51481 (19)	0.0633 (17)	
H45	0.0196	0.0923	0.5311	0.076*	
C46	0.0790 (3)	0.3478 (8)	0.53006 (16)	0.0531 (15)	
H46	0.0735	0.3849	0.5564	0.064*	
C47	0.1208 (2)	0.4700 (8)	0.50523 (16)	0.0447 (14)	
C48	0.1555 (2)	0.6543 (8)	0.51967 (15)	0.0448 (14)	
H48	0.1508	0.6918	0.5460	0.054*	
N3	0.2524 (2)	0.7774 (8)	0.33437 (14)	0.0580 (14)	
N4	0.2909 (2)	0.5966 (7)	0.32609 (14)	0.0588 (13)	
H53	0.2943	0.5528	0.3022	0.071*	
N5	0.2959 (2)	1.2714 (7)	0.50920 (13)	0.0537 (12)	
N6	0.2642 (2)	1.0966 (7)	0.49271 (13)	0.0504 (12)	
H54	0.2680	1.0729	0.4679	0.061*	
O2	0.31792 (19)	0.5390 (6)	0.38926 (11)	0.0695 (12)	
O3	0.21923 (17)	1.0000 (5)	0.55059 (11)	0.0628 (10)	
O5	0.32458 (17)	0.3078 (5)	0.27397 (10)	0.0675 (11)	
H50	0.3139	0.2188	0.2574	0.101*	
O6	0.2452 (2)	0.8363 (6)	0.43242 (11)	0.0657 (11)	
H51	0.2565	0.7649	0.4133	0.099*	
S2	0.17219 (9)	1.1672 (3)	0.35224 (5)	0.0873 (5)	
S3	0.36965 (8)	1.6406 (3)	0.54683 (5)	0.0731 (5)	
C1	0.1105 (3)	0.8520 (9)	0.1823 (2)	0.078 (2)	
H1	0.0887	0.9485	0.1988	0.094*	
C2	0.1095 (3)	0.8610 (10)	0.1431 (2)	0.077 (2)	
H2	0.0854	0.9638	0.1291	0.093*	
C3	0.1482 (2)	0.7003 (8)	0.12479 (16)	0.0556 (16)	
H3	0.1535	0.6864	0.0977	0.067*	
C4	0.1772 (2)	0.5665 (8)	0.15220 (17)	0.0543 (15)	
C5	0.2184 (2)	0.3795 (8)	0.14311 (17)	0.0520 (15)	
H5	0.2278	0.3432	0.1171	0.062*	
C6	0.3083 (3)	−0.0401 (8)	0.18524 (19)	0.0481 (15)	
C7	0.3449 (2)	−0.2287 (8)	0.16877 (15)	0.0398 (13)	
C8	0.3466 (2)	−0.2926 (8)	0.12870 (17)	0.0507 (16)	
C9	0.3838 (3)	−0.4635 (8)	0.11708 (17)	0.0536 (15)	
H9	0.3842	−0.5012	0.0906	0.064*	
C10	0.4212 (3)	−0.5836 (8)	0.14371 (19)	0.0493 (15)	
C11	0.4587 (3)	−0.7676 (10)	0.13163 (19)	0.0676 (18)	
H11	0.4596	−0.8079	0.1053	0.081*	
C12	0.4926 (3)	−0.8815 (9)	0.1585 (2)	0.0762 (19)	
H12	0.5179	−0.9994	0.1506	0.091*	
C13	0.4904 (3)	−0.8241 (10)	0.1989 (2)	0.0767 (19)	
H13	0.5135	−0.9056	0.2174	0.092*	
C14	0.4548 (3)	−0.6509 (9)	0.21041 (18)	0.0634 (18)	
H14	0.4543	−0.6134	0.2369	0.076*	
C15	0.4193 (2)	−0.5292 (8)	0.18397 (18)	0.0463 (15)	
C16	0.3803 (2)	−0.3475 (8)	0.19406 (16)	0.0495 (14)	
H16	0.3792	−0.3078	0.2205	0.059*	
N2	0.27772 (19)	0.0878 (6)	0.15848 (14)	0.0506 (12)	
H2A	0.2806	0.0598	0.1337	0.061*	
O1	0.30712 (18)	−0.0019 (6)	0.22020 (12)	0.0651 (11)	
S1	0.15723 (9)	0.6402 (2)	0.19813 (5)	0.0774 (5)	
N1	0.2415 (2)	0.2660 (7)	0.17149 (14)	0.0509 (12)	
O4	0.31007 (17)	−0.1791 (5)	0.10045 (10)	0.0603 (10)	
H4	0.2714	−0.2252	0.0995	0.090*	

### Atomic displacement parameters (Å^2^)

**Table:**

	*U*^11^	*U*^22^	*U*^33^	*U*^12^	*U*^13^	*U*^23^
C17	0.069 (4)	0.070 (4)	0.093 (3)	0.007 (3)	0.021 (4)	−0.023 (3)
C18	0.099 (5)	0.064 (4)	0.093 (3)	0.024 (3)	0.009 (4)	−0.010 (3)
C19	0.066 (4)	0.054 (4)	0.070 (2)	0.017 (3)	0.009 (3)	−0.004 (3)
C20	0.059 (4)	0.060 (4)	0.072 (3)	0.009 (2)	0.007 (3)	−0.009 (3)
C21	0.069 (4)	0.067 (5)	0.058 (5)	−0.002 (3)	0.001 (4)	−0.006 (4)
C22	0.047 (4)	0.059 (4)	0.043 (4)	−0.010 (3)	0.003 (3)	−0.001 (4)
C23	0.046 (4)	0.056 (4)	0.038 (4)	−0.005 (3)	0.007 (3)	−0.007 (3)
C24	0.050 (4)	0.051 (4)	0.059 (4)	0.001 (3)	0.004 (3)	−0.002 (3)
C25	0.065 (4)	0.056 (4)	0.063 (4)	0.013 (3)	−0.007 (3)	−0.018 (3)
C26	0.044 (4)	0.047 (4)	0.067 (5)	0.001 (3)	0.005 (3)	0.005 (3)
C27	0.070 (5)	0.061 (4)	0.097 (6)	0.002 (3)	−0.012 (4)	0.007 (4)
C28	0.065 (5)	0.069 (5)	0.118 (7)	0.008 (4)	0.013 (5)	0.022 (5)
C29	0.073 (5)	0.107 (7)	0.097 (7)	0.001 (5)	−0.002 (5)	0.058 (6)
C30	0.066 (5)	0.090 (6)	0.077 (5)	−0.004 (4)	0.011 (4)	0.023 (4)
C31	0.047 (4)	0.064 (4)	0.053 (5)	−0.004 (3)	0.000 (3)	0.010 (4)
C32	0.056 (4)	0.066 (4)	0.051 (4)	−0.014 (3)	−0.003 (3)	0.002 (3)
C33	0.056 (4)	0.067 (4)	0.118 (7)	−0.016 (3)	−0.017 (4)	−0.015 (5)
C34	0.070 (5)	0.071 (5)	0.082 (5)	−0.011 (4)	−0.009 (4)	0.011 (4)
C35	0.059 (4)	0.063 (4)	0.057 (4)	−0.004 (3)	0.000 (3)	−0.002 (4)
C36	0.044 (3)	0.048 (4)	0.056 (4)	0.007 (3)	−0.002 (3)	0.006 (3)
C37	0.059 (4)	0.054 (4)	0.045 (4)	−0.001 (3)	0.005 (3)	−0.006 (3)
C38	0.043 (4)	0.053 (4)	0.041 (4)	0.009 (3)	−0.002 (3)	−0.006 (3)
C39	0.043 (3)	0.044 (3)	0.028 (3)	0.004 (2)	0.002 (3)	−0.010 (3)
C40	0.043 (4)	0.043 (4)	0.052 (4)	0.005 (3)	0.007 (3)	0.004 (3)
C41	0.061 (4)	0.067 (4)	0.044 (4)	−0.009 (3)	0.006 (3)	−0.021 (3)
C42	0.044 (4)	0.049 (4)	0.055 (4)	0.004 (3)	−0.006 (3)	−0.006 (3)
C43	0.065 (5)	0.075 (5)	0.065 (5)	−0.025 (3)	0.003 (4)	−0.009 (4)
C44	0.061 (4)	0.066 (5)	0.069 (5)	−0.008 (3)	−0.006 (4)	−0.009 (4)
C45	0.051 (4)	0.065 (5)	0.073 (5)	−0.009 (3)	0.008 (3)	0.009 (4)
C46	0.063 (4)	0.053 (4)	0.043 (4)	0.001 (3)	0.001 (3)	0.003 (3)
C47	0.041 (3)	0.042 (3)	0.051 (4)	0.009 (3)	−0.004 (3)	0.002 (3)
C48	0.057 (4)	0.041 (3)	0.037 (3)	0.018 (3)	0.000 (3)	−0.005 (3)
N3	0.058 (4)	0.050 (3)	0.066 (4)	−0.005 (3)	0.012 (3)	−0.019 (3)
N4	0.073 (4)	0.061 (4)	0.042 (3)	0.002 (3)	0.008 (3)	−0.018 (3)
N5	0.058 (3)	0.045 (3)	0.058 (4)	0.003 (2)	−0.004 (3)	−0.008 (3)
N6	0.064 (3)	0.047 (3)	0.040 (3)	−0.004 (2)	0.005 (3)	−0.012 (2)
O2	0.090 (3)	0.081 (3)	0.037 (3)	0.003 (2)	0.006 (2)	−0.014 (2)
O3	0.074 (3)	0.068 (3)	0.047 (3)	−0.0139 (19)	0.009 (2)	−0.011 (2)
O5	0.086 (3)	0.064 (3)	0.053 (3)	0.020 (2)	−0.007 (2)	−0.018 (2)
O6	0.079 (3)	0.072 (3)	0.045 (3)	−0.016 (2)	0.012 (2)	−0.017 (2)
S2	0.0958 (14)	0.0883 (13)	0.0779 (13)	0.0070 (10)	0.0068 (11)	−0.0152 (11)
S3	0.0846 (13)	0.0801 (12)	0.0545 (11)	−0.0074 (9)	−0.0003 (10)	−0.0064 (10)
C1	0.071 (5)	0.061 (5)	0.103 (7)	0.009 (3)	0.015 (4)	−0.018 (5)
C2	0.058 (4)	0.057 (5)	0.116 (7)	0.006 (3)	−0.003 (4)	−0.002 (5)
C3	0.062 (4)	0.055 (4)	0.050 (4)	−0.001 (3)	0.009 (3)	0.008 (3)
C4	0.053 (4)	0.040 (4)	0.069 (5)	0.000 (3)	0.008 (3)	−0.006 (3)
C5	0.059 (4)	0.044 (4)	0.052 (4)	−0.001 (3)	−0.002 (3)	−0.002 (3)
C6	0.047 (4)	0.043 (4)	0.055 (4)	−0.006 (3)	−0.001 (3)	−0.002 (3)
C7	0.046 (3)	0.041 (3)	0.032 (4)	0.000 (3)	−0.005 (3)	−0.003 (3)
C8	0.044 (4)	0.052 (4)	0.056 (4)	0.008 (3)	−0.006 (3)	0.004 (3)
C9	0.065 (4)	0.046 (4)	0.050 (4)	0.011 (3)	0.004 (3)	−0.016 (3)
C10	0.040 (4)	0.044 (4)	0.063 (5)	0.001 (3)	0.004 (3)	−0.002 (3)
C11	0.061 (4)	0.069 (5)	0.073 (5)	−0.006 (4)	0.001 (4)	−0.003 (4)
C12	0.060 (5)	0.070 (5)	0.098 (6)	0.012 (3)	−0.003 (5)	−0.012 (5)
C13	0.062 (5)	0.066 (5)	0.102 (6)	0.003 (3)	−0.010 (4)	0.014 (4)
C14	0.057 (4)	0.053 (4)	0.080 (5)	0.014 (3)	0.003 (3)	−0.009 (4)
C15	0.038 (4)	0.043 (4)	0.058 (5)	0.003 (3)	−0.002 (3)	0.003 (3)
C16	0.047 (3)	0.057 (4)	0.045 (4)	−0.007 (3)	−0.005 (3)	−0.008 (3)
N2	0.061 (3)	0.049 (3)	0.042 (3)	0.002 (2)	−0.005 (3)	−0.009 (3)
O1	0.084 (3)	0.065 (3)	0.047 (3)	0.014 (2)	−0.002 (2)	−0.011 (2)
S1	0.0790 (12)	0.0771 (12)	0.0762 (12)	0.0032 (10)	0.0082 (10)	−0.0121 (11)
N1	0.057 (3)	0.039 (3)	0.057 (3)	0.007 (2)	0.004 (3)	−0.003 (2)
O4	0.065 (3)	0.069 (3)	0.048 (3)	0.010 (2)	0.006 (2)	−0.012 (2)

### Geometric parameters (Å, °)

**Table:**

C17—C18	1.306 (8)	C42—C47	1.400 (7)
C17—S2	1.695 (6)	C43—C44	1.337 (7)
C17—H17	0.9300	C43—H43	0.9300
C18—C19	1.398 (6)	C44—C45	1.398 (7)
C18—H18	0.9300	C44—H44	0.9300
C19—C20	1.409 (7)	C45—C46	1.355 (6)
C19—H19	0.9300	C45—H45	0.9300
C20—C21	1.442 (7)	C46—C47	1.407 (6)
C20—S2	1.694 (6)	C46—H46	0.9300
C21—N3	1.274 (6)	C47—C48	1.427 (6)
C21—H21	0.9300	C48—H48	0.9300
C22—O2	1.225 (6)	N3—N4	1.392 (5)
C22—N4	1.323 (6)	N4—H53	0.8600
C22—C23	1.510 (7)	N5—N6	1.379 (5)
C23—C32	1.369 (6)	N6—H54	0.8600
C23—C24	1.408 (7)	O5—O1	2.682 (5)
C24—O5	1.382 (6)	O5—H50	0.8199
C24—C25	1.370 (6)	O6—H51	0.8200
C25—C26	1.387 (7)	C1—C2	1.332 (7)
C25—H25	0.9300	C1—S1	1.702 (6)
C26—C31	1.393 (7)	C1—H1	0.9300
C26—C27	1.415 (7)	C2—C3	1.409 (7)
C27—C28	1.336 (8)	C2—H2	0.9300
C27—H27	0.9300	C3—C4	1.376 (6)
C28—C29	1.409 (9)	C3—H3	0.9300
C28—H28	0.9300	C4—C5	1.459 (7)
C29—C30	1.353 (8)	C4—S1	1.673 (6)
C29—H29	0.9300	C5—N1	1.281 (6)
C30—C31	1.387 (7)	C5—H5	0.9300
C30—H30	0.9300	C6—O1	1.211 (6)
C31—C32	1.398 (6)	C6—N2	1.353 (6)
C32—H32	0.9300	C6—C7	1.493 (7)
C33—C34	1.365 (8)	C7—C16	1.334 (6)
C33—S3	1.713 (6)	C7—C8	1.418 (7)
C33—H33	0.9300	C8—C9	1.356 (6)
C34—C35	1.376 (7)	C8—O4	1.396 (5)
C34—H34	0.9300	C9—C10	1.389 (7)
C35—C36	1.365 (6)	C9—H9	0.9300
C35—H35	0.9300	C10—C15	1.409 (7)
C36—C37	1.437 (6)	C10—C11	1.430 (7)
C36—S3	1.682 (5)	C11—C12	1.338 (7)
C37—N5	1.280 (6)	C11—H11	0.9300
C37—H37	0.9300	C12—C13	1.418 (8)
C38—O3	1.245 (6)	C12—H12	0.9300
C38—N6	1.332 (6)	C13—C14	1.350 (7)
C38—C39	1.478 (6)	C13—H13	0.9300
C39—C48	1.373 (6)	C14—C15	1.370 (7)
C39—C40	1.406 (6)	C14—H14	0.9300
C40—C41	1.356 (6)	C15—C16	1.415 (6)
C40—O6	1.370 (5)	C16—H16	0.9300
C41—C42	1.420 (7)	N2—N1	1.397 (5)
C41—H41	0.9300	N2—H2A	0.8600
C42—C43	1.404 (7)	O4—H4	0.8200
			
C18—C17—S2	111.8 (5)	C45—C44—H44	119.8
C18—C17—H17	124.1	C46—C45—C44	121.5 (6)
S2—C17—H17	124.1	C46—C45—H45	119.3
C17—C18—C19	116.8 (6)	C44—C45—H45	119.3
C17—C18—H18	121.6	C45—C46—C47	118.7 (5)
C19—C18—H18	121.6	C45—C46—H46	120.7
C18—C19—C20	107.5 (6)	C47—C46—H46	120.7
C18—C19—H19	126.2	C42—C47—C46	120.1 (5)
C20—C19—H19	126.2	C42—C47—C48	118.9 (5)
C19—C20—C21	128.1 (6)	C46—C47—C48	121.0 (5)
C19—C20—S2	112.6 (4)	C39—C48—C47	121.8 (5)
C21—C20—S2	119.3 (5)	C39—C48—H48	119.1
N3—C21—C20	121.8 (6)	C47—C48—H48	119.1
N3—C21—H21	119.1	C21—N3—N4	114.5 (5)
C20—C21—H21	119.1	C22—N4—N3	121.2 (5)
O2—C22—N4	122.9 (5)	C22—N4—H53	119.4
O2—C22—C23	121.1 (5)	N3—N4—H53	119.4
N4—C22—C23	115.9 (5)	C37—N5—N6	114.5 (5)
C32—C23—C24	117.7 (5)	C38—N6—N5	120.7 (4)
C32—C23—C22	117.2 (5)	C38—N6—H54	119.6
C24—C23—C22	125.1 (5)	N5—N6—H54	119.6
O5—C24—C25	120.6 (5)	C24—O5—O1	103.9 (3)
O5—C24—C23	119.6 (5)	C24—O5—H50	109.6
C25—C24—C23	119.8 (6)	O1—O5—H50	7.9
C24—C25—C26	121.9 (6)	C40—O6—H51	109.5
C24—C25—H25	119.0	C17—S2—C20	91.1 (3)
C26—C25—H25	119.0	C36—S3—C33	91.8 (3)
C25—C26—C31	119.4 (5)	C2—C1—S1	110.9 (5)
C25—C26—C27	121.8 (6)	C2—C1—H1	124.6
C31—C26—C27	118.9 (6)	S1—C1—H1	124.6
C28—C27—C26	120.3 (7)	C1—C2—C3	113.7 (7)
C28—C27—H27	119.9	C1—C2—H2	123.1
C26—C27—H27	119.9	C3—C2—H2	123.1
C27—C28—C29	121.4 (7)	C4—C3—C2	111.2 (6)
C27—C28—H28	119.3	C4—C3—H3	124.4
C29—C28—H28	119.3	C2—C3—H3	124.4
C30—C29—C28	118.3 (7)	C3—C4—C5	125.3 (5)
C30—C29—H29	120.8	C3—C4—S1	111.4 (4)
C28—C29—H29	120.8	C5—C4—S1	123.3 (5)
C29—C30—C31	122.2 (7)	N1—C5—C4	119.0 (5)
C29—C30—H30	118.9	N1—C5—H5	120.5
C31—C30—H30	118.9	C4—C5—H5	120.5
C30—C31—C26	118.9 (6)	O1—C6—N2	122.2 (5)
C30—C31—C32	123.3 (6)	O1—C6—C7	122.1 (5)
C26—C31—C32	117.8 (6)	N2—C6—C7	115.6 (5)
C23—C32—C31	123.4 (6)	C16—C7—C8	116.6 (5)
C23—C32—H32	118.3	C16—C7—C6	117.0 (5)
C31—C32—H32	118.3	C8—C7—C6	126.4 (5)
C34—C33—S3	111.7 (5)	C9—C8—O4	118.8 (5)
C34—C33—H33	124.2	C9—C8—C7	120.9 (5)
S3—C33—H33	124.2	O4—C8—C7	120.2 (5)
C33—C34—C35	111.1 (6)	C8—C9—C10	121.7 (5)
C33—C34—H34	124.4	C8—C9—H9	119.1
C35—C34—H34	124.4	C10—C9—H9	119.1
C36—C35—C34	114.6 (6)	C9—C10—C15	119.1 (5)
C36—C35—H35	122.7	C9—C10—C11	121.7 (6)
C34—C35—H35	122.7	C15—C10—C11	119.1 (5)
C35—C36—C37	124.8 (5)	C12—C11—C10	119.6 (6)
C35—C36—S3	110.7 (4)	C12—C11—H11	120.2
C37—C36—S3	124.5 (4)	C10—C11—H11	120.2
N5—C37—C36	118.6 (5)	C11—C12—C13	120.6 (6)
N5—C37—H37	120.7	C11—C12—H12	119.7
C36—C37—H37	120.7	C13—C12—H12	119.7
O3—C38—N6	120.7 (5)	C14—C13—C12	120.0 (6)
O3—C38—C39	120.8 (5)	C14—C13—H13	120.0
N6—C38—C39	118.5 (5)	C12—C13—H13	120.0
C48—C39—C40	118.8 (5)	C13—C14—C15	121.6 (6)
C48—C39—C38	115.6 (5)	C13—C14—H14	119.2
C40—C39—C38	125.6 (5)	C15—C14—H14	119.2
C41—C40—O6	121.0 (5)	C14—C15—C10	119.2 (5)
C41—C40—C39	120.0 (5)	C14—C15—C16	124.5 (6)
O6—C40—C39	119.0 (5)	C10—C15—C16	116.3 (5)
C40—C41—C42	122.8 (5)	C7—C16—C15	125.3 (5)
C40—C41—H41	118.6	C7—C16—H16	117.4
C42—C41—H41	118.6	C15—C16—H16	117.4
C43—C42—C47	118.8 (5)	C6—N2—N1	119.3 (5)
C43—C42—C41	123.6 (5)	C6—N2—H2A	120.4
C47—C42—C41	117.5 (5)	N1—N2—H2A	120.4
C44—C43—C42	120.6 (6)	C6—O1—O5	143.8 (4)
C44—C43—H43	119.7	C4—S1—C1	92.8 (3)
C42—C43—H43	119.7	C5—N1—N2	112.8 (5)
C43—C44—C45	120.4 (6)	C8—O4—H4	109.5
C43—C44—H44	119.8		
			
S2—C17—C18—C19	2.5 (8)	C38—C39—C48—C47	179.6 (4)
C17—C18—C19—C20	−3.3 (8)	C42—C47—C48—C39	−0.1 (7)
C18—C19—C20—C21	−176.7 (6)	C46—C47—C48—C39	−178.8 (5)
C18—C19—C20—S2	2.7 (6)	C20—C21—N3—N4	178.5 (5)
C19—C20—C21—N3	−176.1 (6)	O2—C22—N4—N3	2.8 (8)
S2—C20—C21—N3	4.5 (8)	C23—C22—N4—N3	179.8 (4)
O2—C22—C23—C32	−10.7 (8)	C21—N3—N4—C22	175.6 (5)
N4—C22—C23—C32	172.2 (5)	C36—C37—N5—N6	−178.1 (4)
O2—C22—C23—C24	170.0 (5)	O3—C38—N6—N5	3.0 (7)
N4—C22—C23—C24	−7.1 (8)	C39—C38—N6—N5	−179.2 (4)
C32—C23—C24—O5	−177.9 (5)	C37—N5—N6—C38	−177.3 (5)
C22—C23—C24—O5	1.4 (8)	C25—C24—O5—O1	21.7 (6)
C32—C23—C24—C25	1.7 (8)	C23—C24—O5—O1	−158.7 (4)
C22—C23—C24—C25	−179.0 (5)	C18—C17—S2—C20	−0.6 (6)
O5—C24—C25—C26	178.6 (5)	C19—C20—S2—C17	−1.3 (5)
C23—C24—C25—C26	−1.0 (8)	C21—C20—S2—C17	178.1 (5)
C24—C25—C26—C31	0.0 (8)	C35—C36—S3—C33	0.0 (4)
C24—C25—C26—C27	179.5 (5)	C37—C36—S3—C33	179.9 (4)
C25—C26—C27—C28	−179.7 (6)	C34—C33—S3—C36	−1.4 (5)
C31—C26—C27—C28	−0.2 (9)	S1—C1—C2—C3	−1.8 (8)
C26—C27—C28—C29	−0.2 (10)	C1—C2—C3—C4	1.5 (8)
C27—C28—C29—C30	−0.6 (10)	C2—C3—C4—C5	177.8 (5)
C28—C29—C30—C31	1.9 (10)	C2—C3—C4—S1	−0.4 (6)
C29—C30—C31—C26	−2.2 (9)	C3—C4—C5—N1	−178.9 (5)
C29—C30—C31—C32	178.4 (6)	S1—C4—C5—N1	−0.9 (7)
C25—C26—C31—C30	−179.1 (5)	O1—C6—C7—C16	−4.1 (8)
C27—C26—C31—C30	1.4 (8)	N2—C6—C7—C16	174.5 (5)
C25—C26—C31—C32	0.2 (8)	O1—C6—C7—C8	176.8 (5)
C27—C26—C31—C32	−179.3 (5)	N2—C6—C7—C8	−4.7 (7)
C24—C23—C32—C31	−1.5 (8)	C16—C7—C8—C9	−1.4 (7)
C22—C23—C32—C31	179.2 (5)	C6—C7—C8—C9	177.7 (5)
C30—C31—C32—C23	179.9 (5)	C16—C7—C8—O4	179.0 (4)
C26—C31—C32—C23	0.5 (8)	C6—C7—C8—O4	−1.9 (8)
S3—C33—C34—C35	2.3 (7)	O4—C8—C9—C10	−179.9 (5)
C33—C34—C35—C36	−2.4 (8)	C7—C8—C9—C10	0.5 (8)
C34—C35—C36—C37	−178.6 (5)	C8—C9—C10—C15	1.3 (8)
C34—C35—C36—S3	1.4 (6)	C8—C9—C10—C11	178.1 (5)
C35—C36—C37—N5	−178.9 (5)	C9—C10—C11—C12	−178.3 (5)
S3—C36—C37—N5	1.1 (7)	C15—C10—C11—C12	−1.6 (8)
O3—C38—C39—C48	1.5 (7)	C10—C11—C12—C13	1.4 (9)
N6—C38—C39—C48	−176.4 (5)	C11—C12—C13—C14	−1.2 (9)
O3—C38—C39—C40	−177.1 (5)	C12—C13—C14—C15	1.0 (9)
N6—C38—C39—C40	5.0 (7)	C13—C14—C15—C10	−1.2 (8)
C48—C39—C40—C41	0.8 (8)	C13—C14—C15—C16	179.2 (5)
C38—C39—C40—C41	179.3 (5)	C9—C10—C15—C14	178.3 (5)
C48—C39—C40—O6	−178.4 (4)	C11—C10—C15—C14	1.5 (8)
C38—C39—C40—O6	0.2 (7)	C9—C10—C15—C16	−2.1 (7)
O6—C40—C41—C42	−178.9 (5)	C11—C10—C15—C16	−178.9 (4)
C39—C40—C41—C42	2.0 (8)	C8—C7—C16—C15	0.6 (7)
C40—C41—C42—C43	179.1 (5)	C6—C7—C16—C15	−178.6 (5)
C40—C41—C42—C47	−3.7 (8)	C14—C15—C16—C7	−179.2 (5)
C47—C42—C43—C44	1.5 (9)	C10—C15—C16—C7	1.2 (8)
C41—C42—C43—C44	178.7 (6)	O1—C6—N2—N1	−2.4 (8)
C42—C43—C44—C45	−1.4 (9)	C7—C6—N2—N1	179.1 (4)
C43—C44—C45—C46	1.0 (9)	N2—C6—O1—O5	−46.8 (9)
C44—C45—C46—C47	−0.8 (8)	C7—C6—O1—O5	131.6 (5)
C43—C42—C47—C46	−1.3 (7)	C24—O5—O1—C6	−136.0 (7)
C41—C42—C47—C46	−178.6 (5)	C3—C4—S1—C1	−0.5 (4)
C43—C42—C47—C48	180.0 (5)	C5—C4—S1—C1	−178.8 (5)
C41—C42—C47—C48	2.7 (7)	C2—C1—S1—C4	1.3 (5)
C45—C46—C47—C42	0.9 (7)	C4—C5—N1—N2	177.6 (4)
C45—C46—C47—C48	179.6 (5)	C6—N2—N1—C5	176.4 (5)
C40—C39—C48—C47	−1.7 (7)		

### Hydrogen-bond geometry (Å, °)

**Table:**

*D*—H···*A*	*D*—H	H···*A*	*D*···*A*	*D*—H···*A*
O6—H51···O2	0.82	2.03	2.771 (5)	149
N6—H54···O6	0.86	1.96	2.641 (5)	135
N2—H2A···O4	0.86	1.96	2.660 (5)	138
N4—H53···O5	0.86	1.90	2.614 (5)	139
O5—H50···O1	0.82	1.87	2.682 (5)	169
O4—H4···O3^i^	0.82	2.40	2.688 (5)	102

Symmetry codes: (i) −*x*+1/2, *y*−3/2, *z*−1/2.
